# Maternal tea consumption and the risk of preterm delivery in urban China: a birth cohort study

**DOI:** 10.1186/s12889-016-3100-3

**Published:** 2016-05-31

**Authors:** Lei Huang, Catherine Lerro, Tao Yang, Jing Li, Jie Qiu, Weitao Qiu, Xiaochun He, Hongmei Cui, Ling Lv, Ruifeng Xu, Xiaoying Xu, Huang Huang, Qing Liu, Yawei Zhang

**Affiliations:** Gansu Provincial Maternity and Child Care Hospital, 143 North Road Qilihe District, Lanzhou, Gansu Province 730050 China; Yale University, School of Public Health, 60 College Street LEPH 440, New haven, CT 06520 USA

**Keywords:** Tea, Preterm, Birth cohort, China

## Abstract

**Background:**

Studies investigating the relationship between maternal tea drinking and risk of preterm birth have reached inconsistent results.

**Methods:**

The present study analyzed data from a birth cohort study including 10,179 women who delivered a singleton live birth were conducted in Lanzhou, China between 2010 and 2012.

**Results:**

Drinking tea (OR = 1.36, 95 % CI: 1.09–1.69), and specifically green (OR = 1.42, 95 % CI: 1.08–1.85) or scented tea (OR = 1.61, 95 % CI: 1.04–2.50), was associated with an increased risk of preterm birth. Drinking tea was associated with both moderate preterm (OR = 1.41, 95 % CI: 1.12–1.79) and spontaneous preterm birth (OR = 1.41, 95 % CI: 1.09–1.83). Risk of preterm birth increased with decreasing age of starting tea drinking (<20 years, OR = 1.60, 95 % CI: 1.17–2.20) and increasing duration (*p* for trend < 0.01). The relationship between tea drinking and preterm birth is modified by both maternal age (*p* < 0.05) and gestational weight gain (*p* < 0.05).

**Conclusions:**

Despite conflicting findings in the previous literature, we saw a significant association with maternal tea drinking and risk of preterm birth in our cohort. More studies are needed both to confirm this finding and to elucidate the mechanism behind this association.

## Background

Tea is among the most widely consumed beverage worldwide [1, 2]. Tea contains tea catechins and phenolic compounds, and has shown a protective effect for risk of ovarian and endometrial cancers [3] and type 2 diabetes in non-pregnant adults [4]. While tea’s beneficial effects have been investigated, potential adverse effects on human reproductive health have also been reported, due to toxicities associated with certain elements in tea and possible contamination [5]. Maternal daily caffeine intake ≥180 mg from tea has been associated with a 38 % increased risk for small for gestational age infants (OR = 1.38, 95 % CI: 1.08–1.76) [6]. At least one drink of tea per day during the periconceptional period was associated with an elevated risk of neural tube defects (OR = 3.4, 95 % CI: 1.4–8.3) [2].

The rapid industrialization in China over the past several decades has caused increased deterioration of the environment, which has brought pollution and contamination to tea [7, 8]. Heavy metal levels in tea were the highest in Chinese samples as compared to those from other countries [9]. While health concerns have been raised for tea consumption during pregnancy [9], up to 21.5 % pregnant women in China are tea consumers [2].

Preterm birth (<37 completed weeks of gestation) is the leading cause of child death [10] and is associated with poor developmental trajectories in infancy [11]. Preterm birth is a multifactorial complex condition whose etiologic influences may act at different times during pregnancy [12], and even prior to the onset of pregnancy [13]. Several epidemiologic studies have investigated tea consumption and preterm birth; however, results have been inconsistent [6, 14–19]. Furthermore, no study has been conducted in Chinese population, where tea consumption is more prevalent and heavy metal contamination is high. In light of the inconsistent results linking tea consumption and risk of preterm birth, as well as a paucity of studies conducted in the Chinese population, we analyzed data from a birth cohort study in Lanzhou, China to examine the hypothesis that tea consumption is associated with an increased risk of preterm birth.

## Methods

A birth cohort study was conducted in the Gansu Provincial Maternity and Child Care Hospital, the largest maternity and child care hospital in Lanzhou, China between 2010 and 2012 [20]. A total of 14,535 eligible women came to the hospital for delivery and 14,359 participated in this study. After excluding women who gave multiple births and/or still birth, 10,179 women were included in the final analysis. All study procedures were approved by the human investigation committees at the Gansu Provincial Maternity and Child Care Hospital and Yale University.

Eligible women were informed of study procedure upon their arrival at the hospital for delivery. After obtaining written consent, trained study interviewers conducted in-person interviews at the hospital using a standardized and structured questionnaire. The majority of women (84 %) were interviewed within three days after delivery, while 16 % of women were interviewed within two days before delivery. Information regarding tea consumption before and during pregnancy was collected. Ever tea drinkers were defined as individuals who drank tea at least three times per week, those who did not fit this description were classified as never tea drinkers [21]. Women were further classified based on whether or not they consumed tea (1) prepregnancy only (consumed tea before pregnancy but not during pregnancy), (2) during pregnancy only (consumed tea during pregnancy but not before pregnancy), (3) prepregnancy (consumed tea before pregnancy, no matter whether or not comsumed tea during pregnancy), (4) during pregnancy (consumed tea during pregnancy, no matter whether or not comsumed tea before pregnancy), and (5) prepregnancy and during pregnancy (consumed tea both before and during pregnancy). Information on types of tea typically consumed (green, black, scented and oolong), age they began drinking tea, and duration of tea consumption (in months) were also collected. The questionnaire also included demographic information and other potential confounding factors including reproductive history, medical conditions and medication use, and environmental and lifestyle factors. Prepregnancy body mass index (BMI) was categorized as underweight (BMI < 18.5 kg/m^2^), normal weight (18.5 kg/m^2^ ≤ BMI < 24 kg/m^2^), and overweight and obese (BMI ≥ 24 kg/m^2^) using the standard of Working Group on Obesity in China [22]. Weight gain during pregnancy was calculated as the difference between prepregnancy and delivery weight. Adequacy of gestational weight gain (GWG) was defined according to the Institute of Medicine GWG recommendations: 12.5–18 kg (prepregnancy BMI < 18.5 kg/m^2^), 11.5–16 kg (BMI 18.5–23.9 kg/m^2^), 7–11.5 kg (BMI 24.0–27.9 kg/m^2^), and 5–9 kg (BMI > 28 kg/m^2^) [23].

Preterm birth was defined as delivery before 37 completed weeks of gestation. The gestational age at delivery was calculated in completed weeks from the first day of the last menstrual period. Preterm birth was divided into moderate preterm birth (32–36 weeks of gestation), very preterm birth (28–31 weeks of gestation), and extremely preterm birth (<28 completed weeks of gestation) according to World Health Organization (WHO) classification [24]. To increase statistical power, we combined very preterm and extremely preterm births into a single group labeled very preterm birth. In addition, preterm births were further classified as either medically indicated or spontaneous [25]. Examples of medically indicated preterm birth include placenta or vasa previa, placenta accreta, placental abruption, prior classical cesarean delivery, uterine rupture or dehiscence, fetal growth restriction, select fetal anomalies, severe preeclampsia, uncontrolled gestational or chronic hypertension, complicated pregestational diabetes, and oligohydramnios.

The relationship between selected characteristcs and tea drinking was analysed using *χ*^2^ test. Multivariate logistic regression models were used to estimate odds ratios (OR) and 95 % confidence intervals (CI) for the association between tea drinking and preterm birth and its clinical subtypes. Models were adjusted for the following potential confounding factors: maternal age (<26, 26–28, 28–31, >31 years), years of education (≤9, 10–15, ≥16), employment status during pregnancy (yes or no), monthly income (≤2000, 2000–4000,>4000 yuan), parity (nulliparous or parous), history of preterm birth (yes or no), hypertension during pregnancy (yes or no), maternal prepregnancy BMI (<18.5, 18.5–23.9, ≥24 kg/m^2^), alcohol consumption (yes or no), and active and/or passive tobacco smoke exposure during pregnancy (yes or no). Additionally, sex of the child, nausea and vomiting during pregancy were included in the models but did not appreciably change the results, therefore they were not included in the final models. All analyses were performed using SAS software, version 9.2 (SAS Institute, Inc., Cary, North Carolina).

## Results

In our study, 7.73 % (787) and 4.38 % (446) women drank tea before and during pregnancy, respectively. 99.6 % of the consumed tea was produced in China. Of the 10,179 singleton live births, 10.01 % (1019) were preterm. Of those 1019 preterm births, 81.75 % (833) were moderate preterm births, 18.25 % (186) were very preterm births, 33.17 % (338) were medically indicated preterm births, and 66.83 % (681) were spontaneous preterm births.

Compared to women who never drank tea, those who drank tea were more likely to be older, less educated, parous, drink alcohol and smoke during pregnancy, and have either ≤2000 or >4000 yuan household income (Table 1). Tea drinkers were also more likely to be diagnosed with hypertention during pregnancy.

**Table 1:** Characteristics of pregnant women depending on never and ever drink tea (≥3 times/week) (*n* = 10 179), Urban China, 2010–2012

Characteristics	Never (*n* = 9303)		Ever (*n* = 876)		*P* value
	*n*	%	*n*	%	
Age, years					<0.0001
< 26	2104	22.62	198	22.6	
26–28	2915	31.33	232	26.48	
28–31	2332	25.07	206	23.52	
> 31	1952	20.98	240	27.40	
Education, years					0.0034
≤ 9	2004	21.54	234	26.71	
10–15	3650	39.23	327	37.33	
≥ 16	3475	37.35	304	34.70	
Missing	174		11		
Employment during pregnancy					0.36
Yes	4812	51.73	439	50.11	
No	4491	48.27	437	49.89	
Monthly income (¥)					0.0003
≤ 2000	2190	23.54	231	26.37	
2000–4000	4365	46.92	349	39.84	
> 4000	1858	19.97	213	24.32	
Missing					
Parity					<0.0001
Nulliparous	6773	72.80	576	65.75	
Parous	2530	27.20	300	34.25	
Pregnancy hypertension disease					0.026
No	8838	95.00	817	93.26	
Yes	465	5.00	59	6.74	
Prepregnancy BMI					0.34
< 18.5	1863	20.03	163	18.61	
18.5–23.9	6146	66.06	573	65.41	
≥ 24	978	10.51	107	12.21	
Missing	316		33		
Alcohol drinking during pregnancy					<0.0001
No	9116	97.99	835	95.32	
Yes	49	0.53	29	3.31	
Missing	138		12		
Active smoke during pregnancy					0.0002
No	9235	99.27	859	98.06	
Yes	68	0.73	17	1.94	
Passive smoke during pregnancy					
No	7623	81.94	614	70.09	<0.0001
Yes	1680	18.06	262	29.91	
Sex of the child					0.51
Boy	4911	52.79	447	51.03	
Girl	4361	46.88	427	48.74	
Missing	31		2		

Abbreviation: *BMI* body mass index

Drinking tea was associated with an increased risk of preterm birth (OR = 1.36, 95 % CI: 1.09–1.69, Table 2). After stratification by subtype, statistically significant associations were seen for moderate (OR = 1.41, 95 % CI: 1.12–1.79) and spontaneous preterm birth (OR = 1.41, 95 % CI: 1.09–1.83). When we examined tea drinking by exposure windows, significant associations were remained for drinking tea before pregnancy, as well as before and during pregnancy, but not for drinking tea during pregnancy only. Significant association was also seen for medically indicated preterm birth with drinking tea during pregnancy ever (OR = 1.76, 95 % CI: 1.13–2.76).

**Table 2:** Associations between tea consumption and risk of preterm birth (*n* = 10 179), Urban China, 2010–2012

Drink tea	Term (*n*)	Preterm (*n* = 1019)		Moderate preterm (*n* = 833)		Very preterm (*n* = 186)		Medically indicated (*n* = 338)		Spontaneous (*n* = 681)	
		No.	OR(95 % CI)	No.	OR(95 % CI)	No.	OR(95 % CI)	No.	OR(95 % CI)	No.	OR(95 % CI)
Never	8403	900	1	733	1	167	1	295	1	605	1
Ever	757	119	1.36(1.09–1.69)	100	1.41(1.12–1.79)	19	1.05(0.64–1.72)	43	1.24(0.85–1.83)	76	1.41(1.09–1.83)
Before pregnancy	680	107	1.39(1.10–1.75)	91	1.47(1.15–1.87)	16	1.01(0.59–1.73)	36	1.24(0.82–1.88)	71	1.49(1.14–1.94)
During pregnancy ever	377	69	1.38(1.04–1.83)	55	1.37(1.00–1.87)	14	1.34(0.75–2.39)	33	1.76(1.13–2.76)	36	1.19(0.83–1.72)
Before pregnancy only	380	50	1.33(0.97–1.83)	45	1.47(1.05–2.05)	5	0.66(0.27–1.65)	10	0.62(0.30–1.28)	40	1.67(1.18–2.36)
Before and during pregnancy	300	57	1.45(1.06–1.98)	46	1.47(1.05–2.06)	11	1.35(0.71–2.58)	26	1.92(1.17–3.15)	31	1.29(0.87–1.92)
During pregnancy only	77	12	1.10(0.57–2.14)	9	1.01(0.48–2.13)	3	1.30(0.38–4.42)	7	1.25(0.48–3.28)	5	0.78(0.30–2.03)

Adjusted for maternal age, educational level, employ status during pregnancy, monthly family income, parity, hypertensive disorder during pregnancy, prepregnancy BMI, alcohol drinking and smoking (active and passive smoking) during pregnancy and history of preterm

Abbreviation: *BMI* body mass index

When the association was examined by duration and age at which women began consuming tea, significant associations were seen for those who had been consuming tea more than 48 months (OR = 1.49, 95 % CI: 1.08–2.04. *P* for trend = 0.008), and for those who began drinking tea before age 20 (OR = 1.60, 95 % CI: 1.17–2.20, Table 3). After stratification by subtypes, similar associations were found for moderate and spontaneous preterm birth.

**Table 3:** Associations between tea consumption and risk of preterm birth by duration and starting age of tea consumption (*n* = 10 179), Urban China, 2010–2012

Drink tea	Term (*n*)	Preterm (*n* = 1019)		Moderate preterm (*n* = 833)		Very preterm (*n* = 186)		Medically indicated (*n* = 338)		Spontaneous (*n* = 68)	
		No.	OR(95 % CI)	No.	OR(95 % CI)	No.	OR(95 % CI)	No.	OR(95 % CI)	No.	OR(95 % CI)
Never	8403	900	1	733	1	167	1	295	1	605	1
Ever											
Duration, month											
≤ 48	321	48	1.24(0.89–1.73)	36	1.16(0.80–1.68)	12	1.52(0.83–2.83)	22	1.61(0.95–2.73)	26	1.08(0.70–1.64)
> 48	304	52	1.49(1.08–2.04)	46	1.62(1.16–2.27)	6	0.84(0.36–1.94)	17	1.14(0.63–2.07)	35	1.65(1.14–2.39)
*P* for trend		0.008		0.0028		0.65		0.26		0.012	
Start drinking tea age, year											
< 20	272	55	1.60(1.17–2.20)	48	1.76(1.26–2.46)	7	0.95(0.43–2.08)	20	1.68(0.97–2.91)	35	1.64(1.13–2.39)
≥ 20	340	46	1.31(0.94–1.83)	39	1.35(0.94–1.94)	7	1.03(0.47–2.26)	14	0.95(0.51–1.80)	32	1.48(1.01–2.17)

Adjusted for maternal age, educational level, employ status, monthly family income, parity, hypertensive disorder complicating pregnancy, prepregnancy BMI, alcohol drinking and smoking (active smoking and passive smoking) during pregnancy and history of preterm

Abbreviation: *BMI* body mass index

Drinking green tea or scented tea consumption was associated with increased risk of preterm birth (OR = 1.42, 95 % CI: 1.08–1.85 and OR = 1.61, 95 % CI: 1.04–2.50, respectively, Table 4). No significant associations were observed for black tea consunption.

**Table 4:** Associations between types of tea consumption and risk of preterm birth (*n* = 10 179), Urban China, 2010–2012

Drink tea	Term (*n*)	Preterm (*n* = 1019)	
		No.	OR(95 % CI)
Never	8403		
Types of tea			
Green tea	502	74	1.42(1.08–1.85)
Black tea	119	17	1.11(0.64–1.91)
Scented tea	140	28	1.61(1.04–2.50)
Before pregnancy			
Green tea	449	67	1.46(1.11–1.93)
Black tea	99	15	1.21(0.68–2.17)
Scented tea	114	24	1.77(1.10–2.84)
During pregnancy			
Green tea	225	38	1.42(0.98–2.05)
Black tea	54	9	1.01(0.47–2.18)
Scented tea	70	15	1.47(0.81–2.65)
Before pregnancy only			
Green tea	257	33	1.41(0.96–2.07)
Black tea	58	8	1.44(0.67–3.13)
Scented tea	62	12	1.87(0.96–3.64)
Before and during pregnancy			
Green tea	172	31	1.53(1.01–2.30)
Black tea	34	7	1.21(0.50–2.91)
Scented tea	44	11	1.71(0.85–3.45)

We further examined whether maternal age and GWG were effect modifiers (Table 5). Significant interactions were observed for tea consumpton and maternal age (*P* for interaction = 0.0208) and GWG (*P* for interaction = 0.0371). Significantly increased risk of preterm birth was associated with tea drinking among women who were aged 30 years or older (OR = 1.87, 95 % CI: 1.36–2.59), and those who had GWG exceeding the recommendations (OR = 1.87, 95 % CI: 1.28–2.73).

**Table 5:** Associations between tea drinking and preterm birth by mother age (*n* = 10 179) and gestational weight gain (*n* = 9752), Urban China, 2010–2012

Characteristics	Never			Ever		
	Term (*n*)	Preterm		Term (*n*)	Preterm	
		No.	OR(95 % CI)		No.	OR(95 % CI)
Maternal age						
< 30	5078	512	1	806	93	1.04(0.81–1.34)
≥ 30	2754	327	1.33(1.05–1.68)	522	87	1.87(1.36–2.59)
Interaction *p* value					0.0208	
Gestational weight gain						
Normal or less normal	3180	507	1	319	58	1.04(0.76–1.43)
Overnormal	4896	311	0.99(0.78–1.26)	405	50	1.87(1.28–2.73)
Interaction *p* value					0.0371	

Adjusted for maternal age, educational level, employ status, monthly family income, parity, hypertensive disorder complicating pregnancy, prepregnancy BMI, alcohol drinking and smoking (active smoking and passive smoking) during pregnancy and history of preterm (additionally, GWG was adjusted for gestational weight gain as a continuous variable)

Abbreviations: *BMI* body mass index, *GWG* gestational weight gain

## Discussion

To the best of our knowledge, this study represents the first to comprehensively examine the associations between tea consumption and preterm birth by various clinical subtypes in a Chinese population. It supports that drinking tea is associated with an increased risk of preterm birth, and that risk varies by preterm birth subtypes.

Caffeine, a xanthine alkaloid, is readily available in tea [26]. Caffeine can readily cross the placental barrier to the fetus [27] and lead to decrease in placental blood supply [28], which may influence fetal growth. Besides, a high plasma total homocysteine level during pregnancy is a factor contributing to preterm birth [29]. Folate plays a crucial role in reducing homocysteine level [30]; however tea catechins inhibit folate metabolism pathway [31] and lead to lower serum folate level during pregnancy [32], which may increase risk of preterm birth. Oxidative stress induced pathological damage plays an important role in preterm birth [33, 34]. Exposure to heavy metals [35] and pesticides [36] in tea leaves can result in abnormally high generation of reactive oxygen species. These reactive oxygen species may lead to irreversible alteration of cellular macromolecules, such as lipids and proteins affecting the normal functioning of mitochondrial membranes, and disrupt reproductive function [37]. Therefore, it is biologically plausible that exposure to tea infusion is associated with an increased risk of preterm birth.

Several studies have investigated the association between tea drinking and preterm birth, however, the results were inconsistent [6, 14–19]. Three studies reported no association between caffeine concentrations from tea consumed during pregnancy and risk of preterm [6, 16, 17]. A Norwegian study reported caffeine consumption from black tea during the first two trimesters was significantly associated with elevated risk of preterm birth (OR = 1.61, 95 % CI: 1.10–2.35) [15]. One study from Connecticut and Massachusetts including 2291 mothers with singleton live births reported that caffeine consumption from tea during the first trimester was associated with significantly increased risk of preterm delivery. However, after adjustment for confounding factors such as parity and smoking, the results were attenuated [18]. Chiaffarino et al. from North Italy [17], Moussally et al. from Quebec [14] and Santos et al. from Brazil [19] reported no significant association between tea consumption during pregnancy and risk of preterm birth. The first two studies used women who did not drink tea during pregnancy as the reference group, without consideration for preconception tea drinking. We similarly saw no association with preterm birth when tea drinkers during pregnancy were compared to those who did not consume tea during pregnancy, regardless of their prepregnancy consumption (data not shown).

In addition to caffeine, heavy metals, pesticides and persistent organic pollutants have also been found in Chinese tea leaves including lead (Pb), chromium (Cr), cadmium (Cd) [1, 38], perfluorooctanoic acid [38], atrazine [39], and dichlorodiphenyltrichloroethane (DDT) [39]. A study from Iran reported that a 1 μg/dl increase in maternal blood lead levels during first trimester led to a 40 % increased risk of preterm birth [40]. A study from Kentucky showed a 26 % increased risk of preterm birth for the highest atrazine exposure group (≥0.08 μg/L) compared with the lowest exposure group (≤0.0015 μg/L) [41]. Perfluorooctanoic acid and DDT have also been linked to elevated risk of preterm delivery [42, 43]. A random sample survey conducted in Beijing, China, showed lead concentrations ranging from 0.20 to 6.35 mg/kg for a local market tea sample [7], concentrations significantly higher than those reported by other countries [44, 45]. An experimental study used 3042 tea samples from south China, discovered pesticide residue concentrations of 30.2–73.4 % tea samples higher than Europe maximum residue limits [39]. The increased risk of preterm delivery associated with tea consumption observed in our study could be due to higher contaminations of tea consumed by our study population.

Tea consumption is a complicated exposure, as different types of tea have different constitutions and contaminations. For example, black tea contains the highest amount of arsenic; oolong tea contains the highest amount of chromium [1], while scented tea contains higher levels of pesticide residue [39]. Existing studies have treated tea as one entity [14, 17] which may obscure associations with preterm birth [42]. We found higher risks of preterm birth for green tea and scented tea consumption. Oolong tea was not analyzed separately in our study due to small numbers.

We observed that tea consumption only during pregnancy was not significantly associated with an increased risk of preterm birth, based on a small numbers of exposed cases. Women who only drank tea during pregnancy had short duration of tea consumption, and likely began drinking tea at older ages. Our study found that the risk of preterm birth was higher for those who started drinking tea before age 20 and for those who had longer duration of tea drinking. In this study, those with younger started age had the longer duration of tea drinking (*r* = −0.72). It is unclear whether the higher risk of preterm associated with starting drinking tea at younger age is due to longer duration of exposure, immaturity of metabolic organ or enzymes might prolong metabolizing harmful elements [6]. Long term consumption of tea may cause lipid-soluble contaminants to bio-accumulate effect in the body [9].

We found that maternal age modified the association between tea consumption and risk of preterm birth. Women who are older would have longer accumulated exposure to environmental pollutants. GWG is correlated with fat retention [46], increased fatty tissues leads to bioaccumulation of environmental contaminants [42] and synergistically increases risk of preterm birth. Future studies are needed to confirm these associations and elucidate the underlying mechanisms.

There were 1.2 million preterm births in China in 2010 [47]. The estimated population attributable fraction is 3.01 % (95 % CI: 0.77 %, 5.61 %), suggesting that 0.77–5.61 % of the preterm birth in the Chinese population could be attributed to maternal tea consumption. Since maternal tea consumption is an easily modifiable risk factor of preterm birth, there are still approximately 10,000 to 70,000 preterm births could be prevented annually by reducing maternal tea consumption before and during pregnancy even though the PAF is relatively small.

A major strength of our study was the relatively large sample size, which allowed us to explore the associations with tea consumption and preterm birth by various clinical subtypes. In addition, detailed information on tea drinking habits allowed us to comprehensively examine associations with tea drinking by exposure time windows, duration, and type of tea. Birth outcomes and maternal complications during pregnancy were obtained from medical records, which minimized potential disease misclassification. Detailed information on potential confounders such as pre-pregnancy BMI, active and passive smoking, and history of preterm delivery were collected and controlled for in our analysis.

Limitations should be considered when interpreting the results. We did not quantify the caffeine concentration from tea drinking, which limited our ability to clarify the relationship between caffeine from tea and preterm birth. Information on exposure was collected through in-person interview before/after delivery; therefore, potential recall bias might exist. However, because in China tea consumption is considered one of healthy lifestyles, and women are not informed to avoid tea consumption during pregnancy, if any recall bias exists, it is likely to be non-differential and would result in an underestimation of the observed association. Lack of quantification of tea consumption prevented us from analyzing more detailed categories of tea drinking. Future studies should collect information on amount of tea consumed per day.

## Conclusions

In conclusion, this study supports the hypothesis that in a Chinese population drinking tea is associated with an increased risk of preterm birth though the underlying etiological mechanism is still unknown. Additionally, the risk of preterm birth might vary by type of tea consumed, age at first tea consumption, and duration of tea consumption. Future studies are needed to explore the potential mechanism by which certain elements in tea, including potential contaminations, influence risk of preterm birth.

## Abbreviations

BMI: body mass index
CI: confidence interval
GWG: gestational weight gain
OR: odds ratio
WHO: World Health Organization
